# Functional Assessment of  α_E_β_7_/E-cadherin Interactions in the Steady State Postnatal Thymus

**DOI:** 10.1080/10446670410001722212

**Published:** 2004-06

**Authors:** Susan E. Prockop, Howard T. Petrie

**Affiliations:** 1 Memorial Sloan-Kettering Cancer Center New York NY USA; 2 Cornell Graduate School of Medical Sciences New York NY USA

## Abstract

T cell differentiation in the thymus depends on sequential interactions between lymphoid progenitors and stromal cells in discrete regions of the cortex. Here, we show that despite α_E_β_7_ expression by a subset of the earliest intrathymic precursors (and E-cadherin expression by thymic stroma), interaction of these elements is not required for proper localization of early progenitors into the cortex, or for successful steady state differentiation. These findings indicate that despite *in vitro* data demonstrating α_E_β_7_ mediated adhesion and proliferation of intrathymic T cell precursor populations, T lymphocyte development can proceed independently of α_E_β_7_/E-cadherin interactions.


*Clinical & Developmental Immunology*, June 2004, Vol. 11 (2), pp. 135–141

Taylor & Francis healthsciences

### Functional Assessment of $\alpha_E\beta_7$/E-cadherin Interactions in the Steady State Postnatal Thymus

SUSAN E. PROCKOP[a,]* and HOWARD T. PETRIE[a,b]

[a]*Memorial Sloan-Kettering Cancer Center, Box 287, 1275 York Avenue, New York, NY 10021, USA;* [b]*Cornell Graduate School of Medical Sciences, New York, NY 10021, USA*

T cell differentiation in the thymus depends on sequential interactions between lymphoid progenitors and stromal cells in discrete regions of the cortex. Here, we show that despite $\alpha_E\beta_7$ expression by a subset of the earliest intrathymic precursors (and E-cadherin expression by thymic stroma), interaction of these elements is not required for proper localization of early progenitors into the cortex, or for successful steady state differentiation. These findings indicate that despite *in vitro* data demonstrating $\alpha_E\beta_7$ mediated adhesion and proliferation of intrathymic T cell precursor populations, T lymphocyte development can proceed independently of $\alpha_E\beta_7$/E-cadherin interactions.

*Keywords*: Cadherin; Integrin; Lymphostromal; T lymphocytes; Thymus

### INTRODUCTION

Like all cells of hematopoietic origin, T lymphocytes must be generated throughout life to replace losses from cellular senescence, trauma, and, in the case of lymphocytes, antigen-driven clonal expansion. During intrathymic residence, multilineage progenitors are induced to undergo a series of differentiative and proliferative events that produce mature T cells. The thymic microenvironment plays a key role in this process, by providing uncommitted progenitors with the signals that induce progressive phases of lineage commitment, proliferative expansion and functional maturity (Anderson *et al.*, 1996). Crucial elements of this microenvironment are a complex extracellular matrix and a heterogeneous array of thymic epithelial cells (TEC) (Owen *et al.*, 1999; Anderson *et al.*, 2000; Savino *et al.*, 2000; Kutlesa *et al.*, 2002). T cell differentiation in the postnatal thymus is intimately linked to migration into and between tissue regions that induce and support various elements of the differentiation process (Petrie, 2003). Although it is widely accepted that stromal cells of the thymic microenvironment including TEC play a pivotal role in thymocyte development, the molecular nature of these interactions is not fully understood.

A number of requirements are implicit in the directional migration of cells within a tissue. These include adhesive interactions between migrating cells and a stable matrix as well as signals that induce directional guidance, promote and limit proliferation and promote differentiation. We have recently shown that the adhesive requirements include $\alpha_4$ integrin-mediated adhesion to a matrix consisting of VCAM-1$^+$ stromal cells (Prockop *et al.*, 2002) and have demonstrated a non-redundant role for CXCR4/CCL12 interactions in directing cortical entry of early T cell precursor populations (Plotkin *et al.*, 2003). In this paper, we address the role of another lymphostromal interaction, between $\alpha_E\beta_7$ and E-cadherin, during differentiation in the postnatal thymus.

Several lines of evidence point to a potential role for $\alpha_E\beta_7$ and E-cadherin interactions in intrathymic T cell development. Cadherins are members of a family of cell adhesion molecules that mediate homotypic interactions critical in embryonic development and in the maintenance of tissue architecture (Kemler, 1992; Angst *et al.*, 2001). Specifically, E-cadherin is known to transmit signals that are important in regulating epithelial cell functions (Kandikonda *et al.*, 1996; Takahashi and Suzuki, 1996; Jankowski *et al.*, 1997). E-cadherin is expressed on TEC, most prominently in the medulla but also in the cortex and in the subcapsular region (Lee *et al.*, 1994). In the human thymus, staining for E-cadherin colocalizes with staining for the medullary TEC marker, TE4 (Kutlesa *et al.*, 2002). These homotypic E-cadherin interactions have been shown to be critical for thymic organogenesis and early thymocyte development in FTOC (Müller *et al.*, 1997). It should be noted that the migratory requirements for fetal and postnatal progenitors cannot be assumed to be the same, since fetal progenitors do not enter the thymus through blood vessels at the CMJ nor do they migrate outward across the cortex during differentiation

*Corresponding author. Tel.: +1-212-639-2150. Fax: +1-212-794-4019. E-mail: prockops@mskcc.org

ISSN 1740-2522 print/ISSN 1740-2530 online © 2004 Taylor & Francis Ltd
DOI: 10.1080/10446670410001722212

(Suniara *et al.*, 2000). In addition, the existence of intact E-cadherin–catenin complexes in the thymus suggests direct functional interactions in TEC (Pan *et al.*, 1998). E-Cadherin cannot be detected by flow cytometry on adult thymocytes (Lee *et al.*, 1994), indicating the potential for other molecules that mediate compensatory functions.

In addition to classic homotypic interactions, E-cadherin undergoes heterophilic interactions as the functional counter-receptor for the integrin $\alpha_E\beta_7$ (Cepek *et al.*, 1994; Higgins *et al.*, 1998; Corps *et al.*, 2001), which is expressed by thymocytes (Lefrancois *et al.*, 1994; Andrew *et al.*, 1996). E-cadherin is the only known binding partner for $\alpha_E\beta_7$, and $\beta_7$ is the only binding partner for αE. In both mouse and human, $\alpha_E\beta_7$ is expressed by early T cell precursors negative for both CD4 and CD8 (DNs) and by a population of medullary CD8 single positive (SP) cells. The expression of $\alpha_E\beta_7$ on T cell precursors and E-cadherin on thymic stroma has been taken as indirect evidence that this receptor-counter receptor pair may participate in thymocyte-thymic stromal cell interactions in the postnatal thymus (Andrew *et al.*, 1996).

Functionally, adhesion of human DN and $CD8^+$ SPs to isolated TECs can be inhibited by antibody to either E-cadherin or $\alpha_E$ integrin (Kutlesa *et al.*, 2002). In this system, signaling through the $\alpha_E\beta_7$ receptor was also demonstrated by proliferation of $CD8^+$ SP cells and by the inhibition of proliferation in the presence of blocking antibody. Additional evidence that $\alpha_E\beta_7$ may function to modulate the response of T lymphocytes to stimulation by epithelial cells is that it is expressed by intraepithelial T lymphocytes of the skin and gut where it is important in localization of T cells within the intestinal epithelium (Andrew *et al.*, 1996; Schon *et al.*, 1999; Agace *et al.*, 2000; Pauls *et al.*, 2001). Integrin $\alpha_E$ deficient mice have been generated and demonstrate the non-redundant role of $\alpha_E\beta_7$/E-cadherin interactions in the localization of mucosal T lymphocytes (Schon *et al.*, 1999). In addition, they have reduced numbers of T lymphocytes in intestinal and vaginal epithelia and develop hyperproliferative inflammatory skin alterations (Schon *et al.*, 2000). This demonstrates that $\alpha_E\beta_7$ contributes to epidermal localization of T cells. However, the intrathymic development of T cells in the $\alpha_E$ deficient mouse has not been formally examined.

We have characterized the expression of $\alpha_E\beta_7$ in T precursor subsets. Functional analyses, including differentiation potential and proliferation studies, were performed to determine the role of $\alpha_E\beta_7$ and its ligand E-cadherin during postnatal thymocyte development. A competitive *in vivo* model such as ours with a minority population of mutant precursors has the potential to demonstrate even a minor defect in T cell development. Surprisingly, we find no absolute requirement for the $\alpha_E\beta_7$/E-cadherin axis in the steady state interactions that T cell progenitors undergo with thymic stroma in the postnatal thymus. Our findings implicate the presence of a compensatory mechanism for these lymphostromal interactions.

### MATERIALS AND METHODS

### Animals

C57BL/6J (CD45.2) and congenic B6/Ly5.2/Cr (CD45.1) mice were purchased from the National Cancer Institute (Frederick, MD, USA). $\alpha_E^{-/-}$ mice backcrossed for 10 generations to C57Bl/6 mice were generously provided by C Parker and obtained from Charles River Laboratories. They were subsequently bred at MSKCC. Typing and confirming the presence of C57BL/6 polymorphisms at 14 cM 5′ and 10 cM 3′ to the *Itgae* locus was performed as described (Bry and Brenner, 2004). The mice were rested for at least 1 week after weaning before use. All mice were housed under pathogen-free conditions in accordance with the procedures outlined in NIH Publication No. 86-23. All experiments involving animals were approved by the Institutional Animal Care and Use Committee of MSKCC.

### Cells

Bone marrow was recovered by flushing cells from femurs and tibias, followed by filtration through mesh. Red blood cells were lysed using 0.15 M $NH_4Cl$/1 mM $KHCO_3$/ 0.1 mM EDTA. Single cell suspensions of thymocytes were prepared by gentle dissociation through wire mesh.

### Microarray Screening

Microarray results were generated as previously described (Plotkin *et al.*, 2003). Briefly, RNA was extracted from purified progenitor populations, labeled and hybridized to the Affymetrix U74A gene chip. For comparison of expression levels between progenitor populations (i.e. between arrays), the mean expression level for all genes characterized as present (i.e. where Wilcoxon rank differences between matched and mismatched probe sets had a null hypothesis significance of $p < 0.04$) was taken. This global mean was then scaled up or down, as appropriate, to an arbitrary value of 500, and all individual gene expression values from that array were adjusted proportionally.

### Flow Cytometric Analyses

All cell suspensions were stained and analyzed at 4°C in mouse-tonicity Hanks' balanced salt solution containing 5% FBS and 0.5% DNase (buffer). Samples of cells prepared as described above were washed with buffer and incubated with optimal concentrations of monoclonal antibodies. Except as indicated, all antibodies were prepared, purified and conjugated on site at MSKCC. Conjugation of antibodies to Alexa dyes was performed as recommended by the manufacturer (Molecular Probes, Eugene, OR). Blocking of non-specific binding was performed with purified Rat IgG and anti-Fc receptor (clone 2.4G2). DN cells from non-chimeric mice were prepared by depletion of $lineage^+$ cells by staining with a cocktail of antibodies (CD3, CD4, CD8, CD19, Gr1, Mac-1 and Ter 119) followed by density particle incubation and density centrifugation (Stem Cell Technologies, Vancouver, BC). Subsequent staining was performed with Alexa488-conjugated anti-αE (clone 2E7), PE-conjugated anti-CD24 (clone M1/69, BD Pharmingen), Alexa633-conjugated anti-CD44 and Alexa660-conjugated anti-CD25. Some samples were additionally stained with biotin labeled anti-ckit (clone ACK-2) and streptavidin PE-Texas Red.

### Construction and Analysis of Stable Bone Marrow Chimeras

Donor bone marrow was prepared by flushing marrow from tibias and fibulas of $\alpha_E^{-/-}$ (Ly5.2/CD45.2) mice, followed by hypotonic lysis of RBC. Recipients for marrow transplantation were sex-matched Ly5.1 (CD45.1) congenic mice. Recipient mice received 6 Gy of gamma irradiation 20 h before transplantation. Irradiated recipients received $3 \times 10^7$ donor cells in total, which were a mixture of mutant CD45.2 donor marrow cells together with syngeneic (CD45.1) marrow. After 5–7 weeks, chimeric mice were sacrificed, and hematopoietic tissues were harvested (thymus, bone marrow, blood, spleen). Similar chimeras were constructed using wild type (Ly5.2) donor marrow. For analysis of CD4/CD8 phenotype of donor and recipient thymocytes, single-cell suspensions were stained with Alexa488 conjugated either to CD45.2 or CD45.1, Thy1.2 PE, CD4 APC and Alexa660-conjugated CD8. For analysis of progenitor thymocyte stages, suspensions were first stained with the cocktail of lineage antibodies described above, followed by PE-Texas Red-conjugated anti-rat IgG, blocking was performed as above with rat IgG and 2.4G2. Following this, cells were stained with the CD45 antibodies described above as well as Thy1.2 PE, Alexa633-conjugated CD44 and alexa660-conjugated CD25. In all cases, DAPI (4′,6-diamidino-2-phenylindole dihydrochloride, Molecular Probes) was used at 0.1 ug/ml for dead cell exclusion; Doublets were eliminated by FSC pulse processing. Samples were acquired on an LSR cytometer (BD Biosciences, San Jose, CA) with modifications as described (Gordon *et al.*, 2003). Post-acquisition analysis was performed using FlowJo software (Tree Star Inc., San Carlos, CA).

### Cell Cycle Analysis

Fixation of stained cells for cell cycle analysis was performed as previously described with DAPI (4′,6-diamidino-2-phenylindole dihydrochloride, Molecular Probes) at 10 μg/ml (Gordon *et al.*, 2003). Cell cycle statistics were generated using the Dean–Jett–Fox algorithm in the FlowJo cell cycle platform.

### RESULTS

### Expression of $\alpha_E$ by Thymic Progenitors

To identify adhesion molecules that might facilitate cell migration and lymphostromal interactions of early progenitors in the post-natal thymus, a number of approaches have been used. In one high throughput method, gene expression analysis was performed using high-density Affymetrix microarrays. RNA template was extracted from defined progenitor stages: these were DN1 ($CD3^-4^-8^-25^-44^{hi}$), DN2 ($CD3^-4^-8^-25^+44^{hi}$), DN3 ($CD3^-4^-8^-25^+44^{lo}$) and pre-DP($CD3^{lo}4^{lo}8^{lo}2544^{lo}$), and hybridized to the chip. Various methods were used to determine gene expression relative to a global mean of all genes on the chip (set arbitrarily to 500; see "Methods" section). One of the candidates that emerged was $\alpha_E$, which pairs with $\beta_7$ to form a heterodimeric protein that recognizes E-cadherin as a ligand (Cepek *et al.*, 1994; Karecla *et al.*, 1995). Expression was nearly undetectable in DN2, DN3 or preDP precursors, but was expressed at high levels on DN1 cells (Fig. 1). $\beta_7$ integrin was more broadly expressed, but unlike $\alpha_E$, $\beta_7$ can form heterodimeric complexes with other integrin chains, including $\alpha_4$, which is also expressed on DN progenitors (Mojcik *et al.*, 1995; Dalmau *et al.*, 1999; Prockop *et al.*, 2002). The limited expression of $\alpha_E$ to the DN1 stage, together with broad expression of $\beta_7$, suggests that DN1 thymocytes are the only early progenitors with

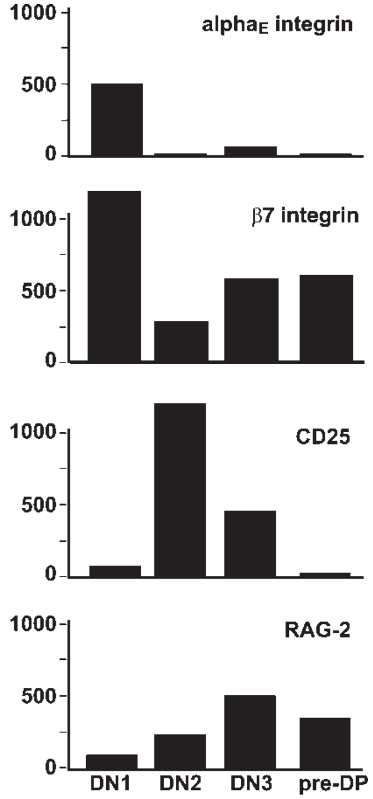

FIGURE 1 Progenitor cells in the thymus express αE integrin. Microarray expression screening was performed on sorted DN subsets. Since $\beta_7$ expression is present in all DN subsets the possibility of $\alpha_E\beta_7$ heterodimers is dictated by expression of $\alpha_E$. $\alpha_E$ is only detectable above baseline in the earliest population of intrathymic T cell precursors. Also shown are relative expression levels of two control genes, CD25 and RAG-1. As expected, these demonstrate highest expression level in DN2 and DN3, respectively. Relative expression levels were calculated by normalizing the mean global expression level for all 12,000-probe sets on the chip to an arbitrary value (in this case, 500).

the capacity to interact via heterotypic binding with stromal cells expressing E-cadherin.

### Surface Expression of $\alpha_E$ by Subsets of Thymic Progenitors

RNA expression data does not necessarily imply surface expression of intact molecules. To confirm expression at the cell surface, $\alpha_E$ integrin staining was performed by flow cytometry using a fluorochrome-conjugated antibody (Fig. 2). On early progenitors, $\alpha_E$ staining was restricted to DN1 cells, consistent with microarray data. In addition, a substantial population of CD8 SP cells expressing $\alpha_E$ was found, consistent with previously published findings. Together, these findings substantiated the possibility that $\alpha_E\beta_7$ integrin might be used to constrain interactions between DN cells and stromal cells expressing the E-cadherin ligand. In particular, the fact that $\alpha_E\beta_7$ was expressed only on the least mature and the most mature cells in the thymus raised the possibility that this adhesion molecule might function in the import and/or export of cells to and/or from the thymus (Lind *et al.*, 2001).

### Stable Bone Marrow Chimeras Demonstrate that $\alpha_E$ Provides no Competitive Advantage to Developing Thymic Progenitors

Although progenitor thymocytes are capable of interacting with E-cadherin expressing TECs *in vitro*, we wanted to determine whether these interactions occurred *in vivo*. Therefore, we sought to confirm the role of $\alpha_E\beta_7$ interactions in the developmental progression of intrathymic progenitors, using stable bone marrow chimeras constructed from donor marrow from $\alpha_E$ deficient ($\alpha_E^{-/-}$) mice or control littermate wild type mice transplanted into sublethally irradiated, wild type, CD45.1-congenic recipients. After return to the steady state (5–7 weeks), thymuses from chimeric animals were removed, and the two lobes were separated. One lobe was used immediately for phenotypic analysis by flow cytometry, and the other was frozen for subsequent histology; in this way, developmental stage could be directly correlated with localization in a single chimeric organ.

Figure 3 shows the results from five mutant chimeras, and three control chimeras. The percent chimerism in thymus, marrow and blood is shown for those constructed from $\alpha_E^{-/-}$ marrow. In all, the degree of chimerism was proportional to the amount of $\alpha_E^{-/-}$ marrow used as original donor marrow. Within the thymus both mutant and wild type marrow resulted in DN, DP, and mature cells of donor origin in normal proportions (Fig. 3) and, other than the Ly5 congenic marker, were essentially indistinguishable from cells of recipient origin. Further characterization of the DN progeny of transplanted donors showed that αE integrin deficiency resulted in no defect in early thymocyte development. When localization studies

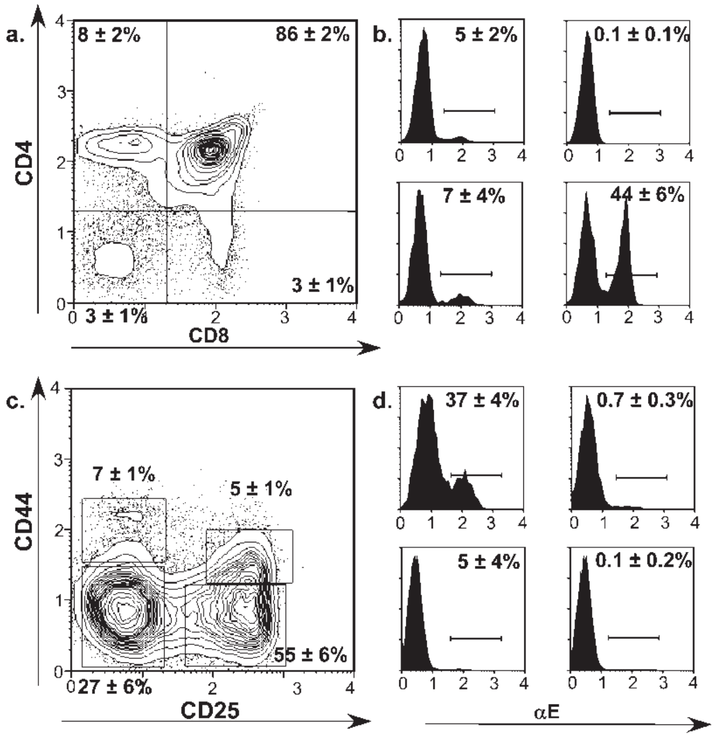

FIGURE 2 Progenitor cells in the thymus stain for cell surface $\alpha_E$ integrin. Wild type thymocytes stained for CD4 and CD8 and $\alpha_E$ (a). Histograms of a*E* staining are gated on events from each quadrant (b) demonstrating a small population of $\alpha_E$ positive CD4 SP cells, and a large population of $\alpha_E$ positive CD8 SP cells. Double negative thymocytes were prepared and stained with $\alpha_E$, CD24, CD44 and CD25 (c). Histograms of $\alpha_E$ staining are shown for each DN population (d). Additional experiments performed with $\alpha_E$, CD24, c-kit, CD44 and CD25 were used to obtain percentages of c-kit positive DN1 progenitors expressing $\alpha_E$. Expression of $\alpha_E$ is demonstrated in the DN1 population where 37% of all cells, and 67% of c-kit positive cells are also positive for $\alpha_E$.

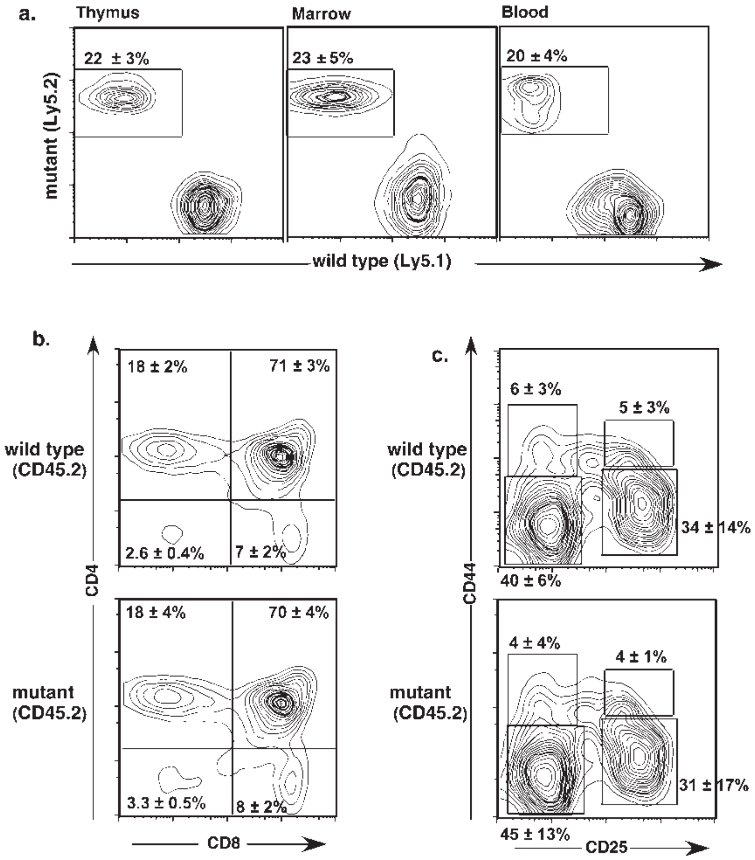

FIGURE 3 Stable hematopoietic chimeras generated from $\alpha_E^{-/-}$ bone marrow demonstrate normal competition by $a_E^{-/-}$ precursors. Staining of chimeric thymus and marrow for $\alpha_E^{-/-}$ cells (Ly5.2) and recipient wild type cells (Ly5.1) is shown. Peripheral blood was additionally stained for and gated on Gr1 as a marker of chimerism in the stem cell compartment (a). Precursors derived from $\alpha_E^{-/-}$ (Ly5.2) and wild type (Ly5.1) bone marrow contribute proportionately to hematopoiesis demonstrating the ability of $\alpha_E\beta_7^{-/-}$ T lymphocyte precursors to compete with wild type. Staining of $\alpha_E^{-/-}$ and wild type chimeric thymuses for Ly5.2, Thy 1.2, CD4 and CD8 is shown gated on $Ly5^+/Thy^+$ events (b) CD4 and CD8 profiles from the donor compartments of $\alpha_E^{-/-}$ and wild type chimeras are normal and indistinguishable from each other. Double negative profiles generated by gating on lineage negative, Ly5/Thy events from $\alpha_E^{-/-}$ chimeras and wild type chimeras (c) are also indistinguishable from each other.

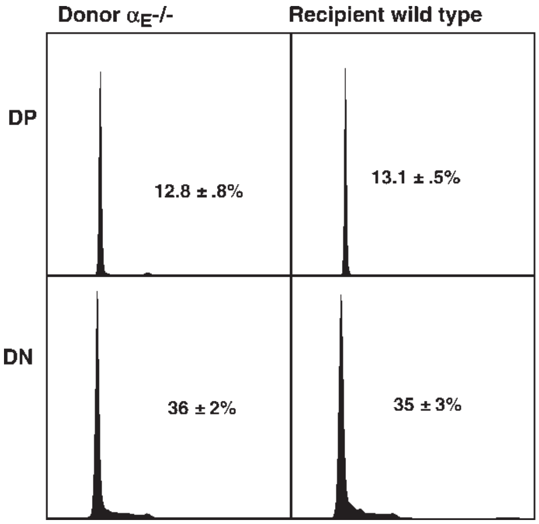

FIGURE 4 Precursors derived from $\alpha_E^{-/-}$ and wild type marrow demonstrate the same proliferative status. $\alpha_E$ and wild type intrathymic precursors were stained with Ly5.2, Thy1.2, CD4 and CD8. After fixation and DAPI staining, cell cycle data was collected for $\alpha_E^{-/-}$ and wild type precursors. Gating on $Thy1^+$, $Ly5.2^+$ ($\alpha_E^{-/-}$ donor) or $Thy^+/Ly5.2^-$ (wild type recipient) allowed analysis of mutant and wild type cell cycle status shown as DAPI histograms for DN and DP populations.

were performed, the hematopoietic progeny of $\alpha_E$ marrow donors were found throughout the thymus (data not shown), consistent with the presence of cells at all developmental stages.

### $\alpha_E$ Expression Confers no Advantage in Proliferative Capacity

Adhesion is a prerequisite event for normal cell proliferation, especially that mediated by integrins (Lind *et al.*, 2001). In chimeric mice, there was no difference between wild type and $\alpha_E^{-/-}$ progenitors in cell cycle distributions at any stage (Fig. 4). These results indicate that in the absence of $\alpha_E$ adhesion and signaling, progenitors recruited from the blood move efficiently into the cortex, proliferate and differentiate and subsequently move into the medulla where they differentiate further. Together, our findings show that despite being present on a discrete subset of early intrathymic progenitors, in addition to some more mature SP cells, $\alpha_E\beta_7$-mediated signaling is not required for mediating cortical localization of progenitors homing to the thymus from the blood or for the success of steady state T cell differentiation.

### DISCUSSION

There is increasing evidence that integrins play a pivotal role in regulating T cell maturation (Mojcik *et al.*, 1995; Andrew *et al.*, 1996; Salomon *et al.*, 1997; Vivinus-Nebot *et al.*, 1999; Savino *et al.*, 2000; Schwartz and Assoian, 2001). Integrins primarily interact with extracellular matrix molecules but also with cellular adhesion molecules like VCAM-1 and E-cadherin. The expression pattern of integrin ligands in the thymus as well as the integrin expression on thymocytes seems to be finely tuned, being responsible for their influence at defined stages of T cell maturation (Schmeissner *et al.*, 2001; Prockop *et al.*, 2002). Developing T cells have to undergo various cycles of proliferation, migration, adhesion and arrest during their differentiation process. Therefore, molecular interactions that coordinate adhesion with proliferation are potentially of high significance for T cell development.

As reviewed in the introduction, a role for $\alpha_E\beta_7$/E-cadherin interactions within the thymus has been indicated by several earlier studies. However, the role of $\alpha_E\beta_7$ in the critical lymphostromal interactions that occur during steady state differentiation has not been characterized. The results presented here document stage specific expression of $\alpha_E\beta_7$ by immature lymphoid precursors in the thymus. However, in contrast to the demonstrated role for homotypic E-cadherin interactions during fetal thymic development, the results here clearly demonstrate that there is no requirement in steady state postnatal thymic lymphopoiesis for $\alpha_E\beta_7$/E-cadherin interactions between T cell precursors and thymic stroma. The fact that this is demonstrated in the face of competition from $\alpha_E\beta_7$ expressing wild type precursors is especially convincing.

It is important to note that the approach described in this paper has been successfully used to demonstrate other mechanisms participating in intrathymic stages of T cell development. We have recently demonstrated that expression of CXCR4 by newly entering progenitors and the presence of CXCL12 throughout the thymic cortex may be sufficient to facilitate the directional migration of early progenitors across the cortex to the capsule. Experiments are now underway to determine the relative roles of other lymphostromal interactions in the transcortical migration process. Nonetheless, it is clear that the $\alpha_E\beta_7$/E-cadherin axis is not critical for these events in the postnatal thymus.

### *Acknowledgements*

The authors wish to thank Drs Christina Parker (Harvard University) and Lynn Bry (Brigham & Women's Hospital) for providing heterozygote αE deficient mice. We also thank Drs Ana Lepique, Sahba Tabrizifard and Willem Van Ewijk for helpful discussions and comments regarding the manuscript. This work was supported by PHS grants AI33940 and AI53739 (to H.T. Petrie) and CA08748 (to MSKCC). S.E. Prockop was supported by CA09512.

### *References*

Agace, W.W., Higgins, J.M., Sadasivan, B., Brenner, M.B. and Parker, C.M. (2000) "T-lymphocyte–epithelial-cell interactions: integrin alpha(E)(CD103)beta(7), LEEP-CAM and chemokines", *Curr. Opin. Cell Biol.* **12**, 563–568.

Anderson, G., Moore, N.C., Owen, J.J. and Jenkinson, E.J. (1996) "Cellular interactions in thymocyte development", *Annu. Rev. Immunol.* **14**, 73–81.

Anderson, G., Harman, B.C., Hare, K.J. and Jenkinson, E.J. (2000) "Microenvironmental regulation of T cell development in the thymus", *Semin. Immunol.* **12**(5), 457–464.

Andrew, D.P., Rott, L.S., Kilshaw, P.J. and Butcher, E.C. (1996) "Distribution of $\alpha_4\beta_7$ and $\alpha_E\beta_7$ integrins on thymocytes, intestinal epithelial lymphocytes and peripheral lymphocytes", *Eur. J. Immunol.* **26**, 897–905.

Angst, B.D., Marcozzi, C. and Magee, A.I. (2001) "The cadherin superfamily: diversity in form and function", *J. Cell Sci.* **114**, 629–641.

Bry, L. and Brenner, M.B. (2004) "Critical role of T cell-dependent serum antibody, but not the gut-associated lymphoid tissue, for surviving acute mucosal infection with *Citrobacter rodentium*, an attaching and effacing pathogen", *J. Immunol.* **172**(1), 433–441.

Cepek, K.L., Shaw, S.K., Parker, C.M., Russell, G.J., Morrow, J.S., Rimm, D.L. and Brenner, M.B. (1994) "Adhesion between epithelial cells and T lymphocytes mediated by E-cadherin and the $\alpha_E\beta_7$ integrin", *Nature* **372**, 190–193.

Corps, E., Carter, C., Karecla, P., Ahrens, T., Evans, P. and Kilshaw, P. (2001) "Recognition of E-cadherin by integrin alpha(E)beta(7): requirement for cadherin dimerization and implications for cadherin and integrin function", *J. Biol. Chem.* **276**(33), 30862–30870.

Dalmau, S.R., Freitas, C.S. and Savino, W. (1999) "Upregulated expression of fibronectin receptors underlines the adhesive capability of thymocytes to thymic epithelial cells during the early stages of differentiation: lessons from sublethally irradiated mice", *Blood* **93**(3), 974–990.

Gordon, K.M., Duckett, L., Daul, B. and Petrie, H.T. (2003) "A simple method for detecting up to five immunofluorescent parameters together with DNA staining for cell cycle or viability on a benchtop flow cytometer", *J. Immunol. Methods* **275**(1–2), 113–121.

Higgins, J.M., Mandlebrot, D.A., Shaw, S.K., Russell, G.J., Murphy, E.A., Chen, Y.T., Nelson, W.J., Parker, C.M. and Brenner, M.B. (1998) "Direct and regulated interaction of integrin $\alpha_E\beta_7$ with E-cadherin", *J. Cell Biol.* **140**, 197–210.

Jankowski, J.A., Bedford, F.K. and Kim, Y.S. (1997) "Changes in gene structure and regulation of E-cadherin during epithelial development, differentiation, and disease", *Prog. Nucleic Acid Res.* **57**, 187.

Kandikonda, S., Oda, D., Niederman, R. and Sorkin, B.C. (1996) "Cadherin-mediated adhesion is required for normal growth regulation of human gingival epithelial cells", *Cell Adhes. Commun.* **4**(1), 13–24.

Karecla, P.I., Bowden, S.J., Green, S.J. and Kilshaw, P.J. (1995) "Recognition of E-cadherin on epithelial cells by the mucosal T cell integrin alpha M290 beta 7 (alpha E beta 7)", *Eur. J. Immunol.* **25**(3), 852–856.

Kemler, R. (1992) "Classical adherins", *Semin. Cell Biol.* **3**, 149–155.

Kutlesa, S., Wessels, J.T., Speiser, A., Steiert, I., Muller, C.A. and Klein, G. (2002) "E-cadherin-mediated interactions of thymic epithelial cells with CD103+ thymocytes lead to enhanced thymocyte cell proliferation", *J. Cell Sci.* **115**(Pt 23), 4505–4515.

Lee, M.G., Sharrow, S.O., Farr, A.G., Singer, A. and Udey, M.C. (1994) "Expression of the homotypic adhesion molecule E-cadherin by immature murine thymocytes and thymic epithelial cells", *J. Immunol.* **152**, 5653–5565.

Lefrancois, L., Barrett, T.A., Havran, W.L. and Puddington, L. (1994) "Developmental expression of the IEL $\beta_7$ integrin on T cell receptor and T cell receptor $\beta$ T cells", *Eur. J. Immunol.* **24**, 635–640.

Lind, E.F., Prockop, S.E., Porritt, H.E. and Petrie, H.T. (2001) "Mapping precursor movement through the postnatal thymus reveals specific microenvironments supporting defined stages of early lymphoid development", *J. Exp. Med.* **194**(2), 127–134.

Mojcik, C.F., Salomon, D.R., Chang, A.C. and Shevach, E.M. (1995) "Differential expression of integrins on human thymocyte subpopulations", *Blood* **86**(11), 4206–4217.

Müller, K.M., Luedecker, C.J., Udey, M.C. and Farr, A.G. (1997) "Involvement of E-cadherin in thymus organogenesis and thymocyte maturation", *Immunity* **6**, 257–264.

Owen, J.J., McLoughlin, D.E., Suniara, R.K. and Jenkinson, E.J. (1999) "Cellular and matrix interactions during the development of T lymphocytes", *Braz. J. Med. Biol. Res.* **32**(5), 551–555.

Pan, C.C., Ho, D.M., Chen, W.Y., Chiang, H., Fahn, H.J. and Wang, L.S. (1998) "Expression of E-cadherin and alpha- and beta-catenins in thymoma", *J. Pathol.* **184**(2), 207–211.

Pauls, K., Schön, M., Kubitza, R.C., Homey, B., Wiesenborn, A., Lehmann, P., Ruzicka, T., Parker, C.M. and Schön, M.P. (2001) "Role of integrin $\alpha_E$(CD103)$\beta_7$ for tissue-specific epidermal localization of CD8 + T lymphocytes", *J. Investig. Dermatol.* **117**, 569–575.

Petrie, H.T. (2003) "Cell migration and the control of post-natal T-cell lymphopoiesis in the thymus", *Nat. Rev. Immunol.* **3**(11), 859–866.

Plotkin, J., Prockop, S.E., Lepique, A. and Petrie, H.T. (2003) "Critical role for CXCR4 signaling in progenitor localization and T cell differentiation in the postnatal thymus", *J. Immunol.* **171**(9), 4521–4527.

Prockop, S.E., Palencia, S., Ryan, C.M., Gordon, K., Gray, D. and Petrie, H.T. (2002) "Stromal cells provide the matrix for migration of early lymphoid progenitors through the thymic cortex", *J. Immunol.* **169**(8), 4354–4361.

Salomon, D.R., Crisa, L., Mojcik, C.F., Ishii, J.K., Klier, G. and Shevach, E.M. (1997) "Vascular cell adhesion molecule-1 is expressed by cortical thymic epithelial cells and mediates thymocyte adhesion. Implications for the function of alpha4beta1 (VLA4) integrin in T-cell development", *Blood* **89**(7), 2461–2471.

Savino, W., Dalmau, S.R. and Dealmeida, V.C. (2000) "Role of extracellular matrix-mediated interactions in thymocyte migration", *Dev. Immunol.* **7**, 2779–2791.

Schmeissner, P.J., Xie, H., Smilenov, L.B., Shu, F. and Marcantonio, E.E. (2001) "Integrin functions play a key role in the differentiation of thymocytes *in vivo*", *J. Immunol.* **167**(7), 3715–3724.

Schon, M.P., Arya, A., Murphy, E.A., Adams, C.M., Strauch, U.G., Agace, W.W., Marsal, J., Donohue, J.P., Her, H., Beier, D.R., Olson, S., Lefrancois, L., Brenner, M.B., Grusby, M.J. and Parker, C.M. (1999) "Mucosal T lymphocyte numbers are selectively reduced in integrin alpha E (CD103)-deficient mice", *J. Immunol.* **162**(11), 6641–6649.

Schon, M.P., Schon, M., Warren, H.B., Donohue, J.P. and Parker, C.M. (2000) "Cutaneous inflammatory disorder in integrin alphaE (CD103)-deficient mice", *J. Immunol.* **165**(11), 6583–6589.

Schwartz, M.A. and Assoian, R.K. (2001) "Integrins and cell proliferation: regulation of cyclin-dependent kinases via cytoplasmic signaling pathways", *J. Cell Sci.* **114**(Pt 14), 2553–2560.

Suniara, R.K., Jenkinson, E.J. and Owen, J.J. (2000) "An essential role for thymic mesenchyme in early T cell development", *J. Exp. Med.* **191**(6), 1051–1056.

Takahashi, K. and Suzuki, K. (1996) "Density-dependent inhibition of growth involves prevention of EGF receptor activation by E-cadherin-mediated cell–cell adhesion", *Exp. Cell Res.* **226**(1), 214–222.

Vivinus-Nebot, M., Ticchioni, M., Mary, F., Hofman, P., Quaranta, V., Rousselle, P. and Bernard, A. (1999) "Laminin 5 in the human thymus: control of T cell proliferation via alpha6beta4 integrins", *J. Cell Biol.* **144**(3), 563–574.

